# CYP26A1 Is a Novel Cancer Biomarker of Pancreatic Carcinoma: Evidence from Integration Analysis and *In Vitro* Experiments

**DOI:** 10.1155/2022/5286820

**Published:** 2022-06-06

**Authors:** Yi Yu, Yunxing Wang, Yufeng Zou, Yuan Yu

**Affiliations:** ^1^Department of Pediatrics, Ruijin Hospital, Shanghai Jiao-Tong University, School of Medicine, Xi Wang Road 999, Shanghai, China 201801; ^2^Department of Emergency, Ruijin Hospital, Shanghai Jiao-Tong University School of Medicine, Xi Wang Road 999, Shanghai, China 201801; ^3^Department of General Surgery, Shanghai Fifth People's Hospital, Fudan University, Shanghai 200240, China

## Abstract

**Background:**

CYP26A1 has been reported in multiple cancers. However, the role of CYP26A1 in pancreatic cancer (PC) has not been explored.

**Method:**

The public data used for this study was obtained from The Cancer Genome Atlas (TCGA), Gene Expression Omnibus (GEO), and Cancer Cell Line Encyclopedia (CCLE) cell lines. CCK8, colony formation, and EdU assay were used to assess the proliferation ability of cancer cells. Transwell and wound healing assays were used to evaluate the invasion and migration ability of cancer cells. qRT-PCR and western blot assays were used to analyze the RNA and protein level of genes. Survival package was used for prognosis analysis. Gene Set Enrichment Analysis (GSEA) was used to identify biological pathway differences between two groups. ssGSEA analysis was used to quantify the immune microenvironment in PC tissue. GDSC and TIDE analyses were used for sensitivity analysis of chemotherapy and immunotherapy.

**Results:**

Our results showed that CYP26A1 was overexpressed in PC tissue and cell lines. Meanwhile, metastatic PC cell lines tend to have a higher CYP26A1 level compared with the primary PC cell lines based on CCLE data. Moreover, CYP26A1 was associated with worse clinical features. Also, we found that CYP26A1 had a satisfactory efficiency in predicting overall survival, disease-specific survival, and progression-free interval of PC patients, independent of other clinical features. *In vitro* experiments indicated that CYP26A1 could significantly facilitate the proliferation, invasion, and migration ability of PC cells. GSEA showed that the pathways of angiogenesis, E2F target, MYC target, mTORC signaling, G2M checkpoint, and epithelial-mesenchymal transition were activated in high CYP26A1 patients. Immune infiltration analysis showed that CYP26A1 was positively correlated with macrophages, Th1 cells, and Treg cells, but negatively correlated with Th17 cells. TIDE analysis showed that non_responder patients had a higher CYP26A1 level compared with predicted responder patients of immunotherapy. Drug sensitivity analysis and assay showed that CYP26A1 could increase the chemotherapy sensitivity of gemcitabine.

**Conclusions:**

In summary, CYP26A1 promotes PC progression and is a novel biomarker of PC, with potential for clinical application.

## 1. Introduction

Pancreatic cancer (PC) is one of the most common digestive system malignant globally, with an extremely high mortality rate [1]. Surgical resection combined with neoadjuvant/adjuvant therapy is the mainstay choice for PC patients. However, the 5-year survival rate of PC patients is still less than 10%, independent of the disease stage [2]. Although approximately 40-50% of PC patients presented metastasis-free locally advanced disease, the local control (LC) and overall survival (OS) rates of them remain barren [3]. Therefore, the identification of novel diagnosis and prognosis biomarkers with clinical application potential is crucial in PC research.

Cytochrome P450 26A1 (CYP26A1) is a member of the cytochrome P450 superfamily of enzymes, involved in retinoic acid (RA) metabolism and synthesis of cholesterol, steroids, and other lipids [4]. Currently, CYP26A1 has been reported in multiple cancers. For instance, Chen and their colleagues showed that CYP26A1 was associated with the elevated risk of oral and pharyngeal cancers [5]. Osanai and Lee indicated that CYP26A1 could upregulate fascin, thereby promoting the malignant behavior of breast carcinoma cells [6]. Moreover, Osanai and Lee also revealed that overexpressed CYP26A1 might contribute to the development and progression of cervical malignancies and squamous neoplasia of the head and neck [7]. Patel and their colleagues showed that the RA metabolism inhibitor of RAMBA could significantly hamper the growth of prostate and breast cancer cells [8]. In the vertebrate model, Shelton and their colleagues found that the CYP26A1 was upregulated in adenomatous polyposis coli through a WNT-dependent manner, which could promote colon tumor development [9]. However, there is still limited research focused on the role of CYP26A1 in PC.

In this study, we firstly explored the expression pattern of CYP26A1 based on the open-accessed data obtained from The Cancer Genome Atlas (TCGA) database. The result showed that CYP26A1 was associated with worse clinical features and prognosis. Meanwhile, CYP26A1 had a good prediction efficiency in predicting the overall survival (OS), disease-specific survival (DSS), and progression-free interval (PFI) of PC patients. *In vitro* experiments showed that CYP26A1 was upregulated in PC cells and could remarkably facilitate the proliferation, invasion, and migration of PC cells. Further, gene set enrichment analysis (GSEA) was performed to identify the biological pathway differentially activated in the CYP26A1 low and high group. Immune infiltration analysis was performed with single sample GSEA (ssGSEA) algorithm to explore the effect of CYP26A1 on the immune microenvironment. Moreover, we also found that CYP26A1 might affect the chemosensitivity of PC patients.

## 2. Materials and Methods

### 2.1. Data Acquisition

The open-accessed transcript and clinical information were downloaded from the TCGA database (https://www.cancer.gov/tcga, pancreatic cancer, TCGA-PAAD). In detail, the transcript data was “FPKM” form, and the clinical data was “xml” form. Probe annotation was conducted based on the reference file “Homo_sapiens.GRCh38.gtf”. Limma package in R environment was used for data preprocessing [10]. Survival package in R environment was used to perform Kaplan-Meier survival analysis. CYP26A1 expression pattern in PC cell lines was obtained from Cancer Cell Line Encyclopedia (CCLE) database (https://sites.broadinstitute.org/ccle).

### 2.2. Pathway Enrichment Analysis

GSEA analysis was performed to explore the underlying biological difference between high and low CYP26A1 patients [11]. ClusterProfiler and fgsea package in R environment were used to conduct GSEA analysis [12]. Hallmark gene set was set as the reference file. Gene sets with NOM *P* value < 0.05 were considered significant.

### 2.3. Immune Infiltration Analysis

The immune microenvironment was quantified using ssGSEA algorithm, an extension of the GSEA algorithm [11]. The ssGSEA algorithm quantities 24 immune cell populations in individual tumor samples based on the gene expression patterns.

### 2.4. Drug Sensitivity and Immunotherapy Analysis

The association between CYP26A1 and drug sensitivity was explored based on the GDSC Genomics of Drug Sensitivity in Cancer (GDSC) database (https://www.cancerrxgene.org) [13]. Tumor immune dysfunction and exclusion (TIDE) analysis (http://tide.dfci.harvard.edu/login/) was performed to explore the underlying effect of CYP26A1 on PC immunotherapy [14].

### 2.5. Cell Lines and Tissue

PC cell lines (SW1990, PANC-1, CAPAN-1, and JF305) and normal pancreatic duct epithelial cells (HPDEC-C7) were laboratory stocks. Four paired pancreatic cancer and adjacent tissue were collected from Shanghai Ruijin Hospital. All the patients signed a written informed consent form, which was approved by the Ethics Committee of Ruijin Hospital and performed according to the Declaration of Helsinki.

### 2.6. Quantitative Real-Time PCR (qRT-PCR)

Total RNA was extracted using RNA simple total RNA kit (TIANGEN, Beijing, China) following the protocol, which was then reverse transcribed to cDNA. qRT-PCR was performed based on the SYBR Green system. The primers used were as follows: CYP26A1, forward, 5′-GATTCATGCTGCCTCACCCA-3′; reverse, 5′-GAAGCTGCCAGTCACAATGC-3′, GAPDH, forward, 5′-GCAAATTCCATGGCACCGT-3′; reverse, 5′-TCGCCCCACTTGATTTTGG-3′.

### 2.7. Western Blot

Total proteins were extracted using a total protein extraction kit (Beyotime, Jiangsu, China). Western blot was performed according to the standard process and transferred to PVDF membranes. The primary antibodies were purchased from the Abcam and Proteintech, including CYP26A1 monoclonal antibody (Abcam, 1 : 5000), E-cadherin polyclonal antibody (Proteintech, 1 : 5000), N-cadherin polyclonal antibody (1 : 2000), Vimentin polyclonal antibody (1 : 5000), and GAPDH polyclonal antibody (1 : 10000).

### 2.8. Cell Transfection

Cell transfection was performed using the Lipofectamine 2000 transfection kits (Invitrogen, Carlsbad, CA, USA) following the protocol. CYP26A1 shRNA and control plasmid were purchased from Genepharma (Suzhou, China). The following shRNA sequences were used: shRNA1: 5′-GAGGAAGTTCCTGCAGATGAA-3′; shRNA2: 5′-CTGAAGAGTAAGGGTTTACTT-3′; shRNA3: 5′-GACTTTATATTTAATTTCTAA-3′.

### 2.9. Cell Proliferation Assay

The proliferation ability of PC cells was assessed using CCK8 and colony formation assay. CCK8 assay was performed using a CCK8 kit following the protocol. For the colony formation assay, cells were seeded into a six-plate well with 500 cells per well and then cultured in conventional conditions for 14 days. The culture medium was changed every four days. Finally, cells were fixed with formaldehyde and stained with 0.5% crystal violet. EdU assay was performed using the EdU kit (Beyotime, Biotechnology) following the protocol.

### 2.10. Drug Sensitivity Assays

Cells were plated into a 96-well plate with 3000 cells per well (200 *μ*L medium containing 10% fetal bovine serum). After the cells adhered to the wall, the cells were incubated with different concentrations of gemcitabine for 48 hours. CCK8 assay was used to test the cytotoxicity of gemcitabine (Selleck, S1714). Following the protocol, the viability of cells was determined by ELISA using 450 nm optical density (OD) readings. The half-maximal inhibitory concentration (IC50) was calculated using GraphPad Prism 6.0 (GraphPad Software).

### 2.11. Transwell Assay

Transwell assay was performed using 8-*μ*m pore transwell chambers. In detail, the upper chamber was added with 5 × 10^4^ PC cells and 200 *μ*L medium (10% BSA). The lower chamber was added with 600 *μ*L medium without BSA. After that for 24 hours, the cells were fixed with formaldehyde and stained with 0.5% crystal violet.

### 2.12. Wound Healing Assay

PC cells were seeded into a six-plate well and cultured to 90% confluency. A 10 *μ*L tip was used to make scratches. Then, serum-free medium was added. Cultures were photographed at 0, 24, and 48 hours time points.

### 2.13. Statistical Analysis

GraphPad Prism 8 and R software v4.0.0 were used for statistical analysis. *P* value is two-sided, and less than 0.05 was considered statistically significant. Student *t*-test was used for variables with normal distribution. Kruskal–Wallis test was used for variables with nonnormal distribution.

## 3. Results

### 3.1. CYP26A1 Is Upregulated in PC and Associated with Worse Clinical Features

**Figure 1:**
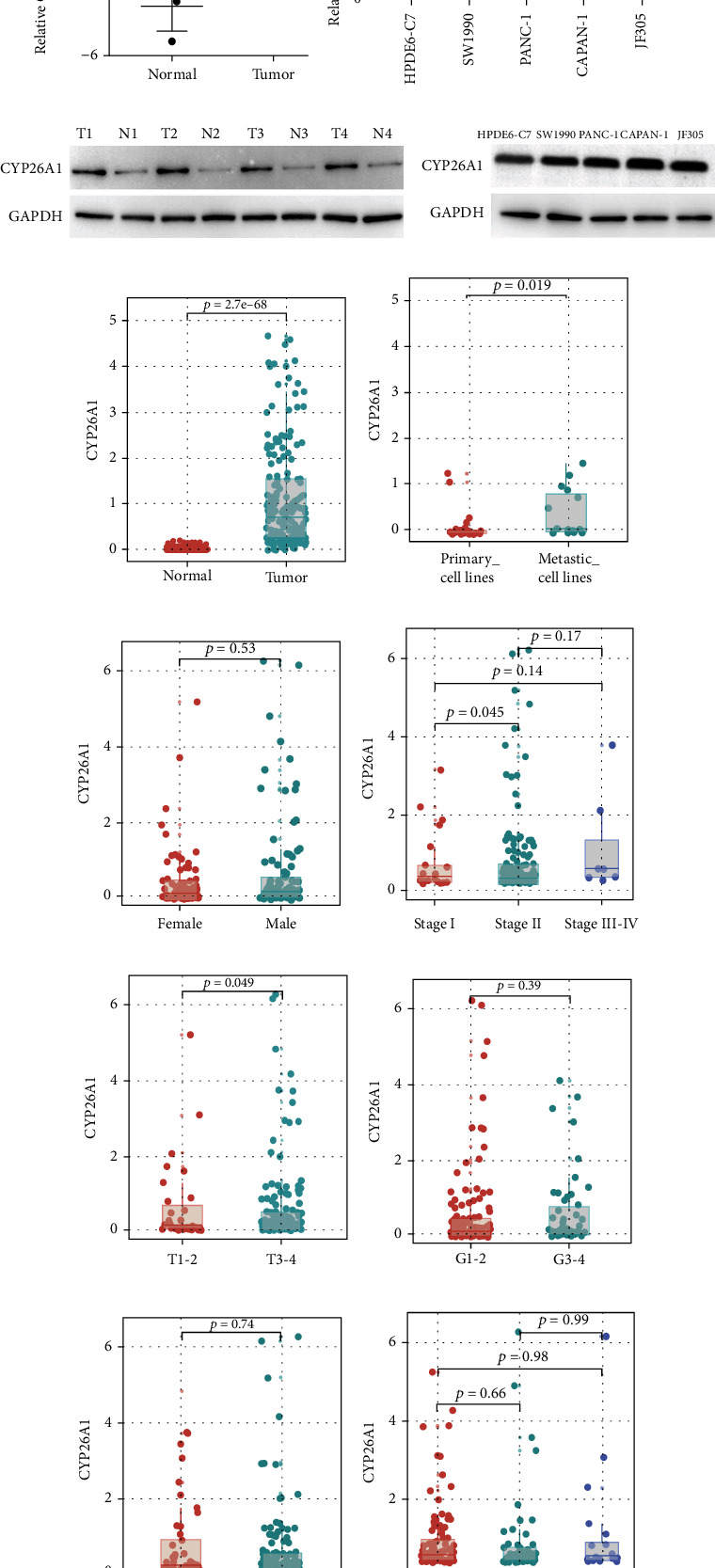
CYP26A1 was upregulated in PC and associated with worse clinical characteristics. Notes: (a) CYP26A1 was upregulated in PC tissue based on four paired PC and adjacent tissue. (b) CYP26A1 was upregulated in PC cell lines compared with normal cell line. (c, d) The protein level of CYP26A1 was upregulated in PC tissue and cell lines. (e) CYP26A1 was upregulated in PC tissue based on TCGA data. (f) CYP26A1 was overexpressed in the metastatic cell lines of PC compared with the primary cell lines. (g)–(l) The expression pattern of CYP26A1 in different groups.

The result in the real world showed that CYP26A1 was overexpressed in PC tissue (Figure 1(a)). Meanwhile, a higher CYP26A1 expression level was also found in PC cell lines compared with the normal HPDE6-C7 cells (Figure 1(b)). The same trend was also observed at the protein level (Figures 1(c) and 1(d)). Based on the open-accessed data obtained from the TCGA database, we also noticed a higher expression pattern of CYP26A1 in PC tissue (Figure 1(e)). Meanwhile, we found that the metastatic PC cell lines tend to have a higher CYP26A1 level compared with the primary PC cell lines, indicating that CYP26A1 might be involved in PC metastasis (Figure 1(f)). No significant difference of CYP26A1 level was found between male and female patients (Figure 1(g)). Clinical correlation analysis showed that stage II patients had a higher CYP26A1 level than stage I patients (Figure 1(h)); T3-4 stage patients had a higher CYP26A1 level than T1-2 stage patients (Figure 1(i)). However, no significant difference was observed in different N stage, grade, and new tumor events, partly due to sample bias (Figures 1(j)–1(l)).

### 3.2. CYP26A1 Is a Prognosis Biomarker of PC

**Figure 2:**
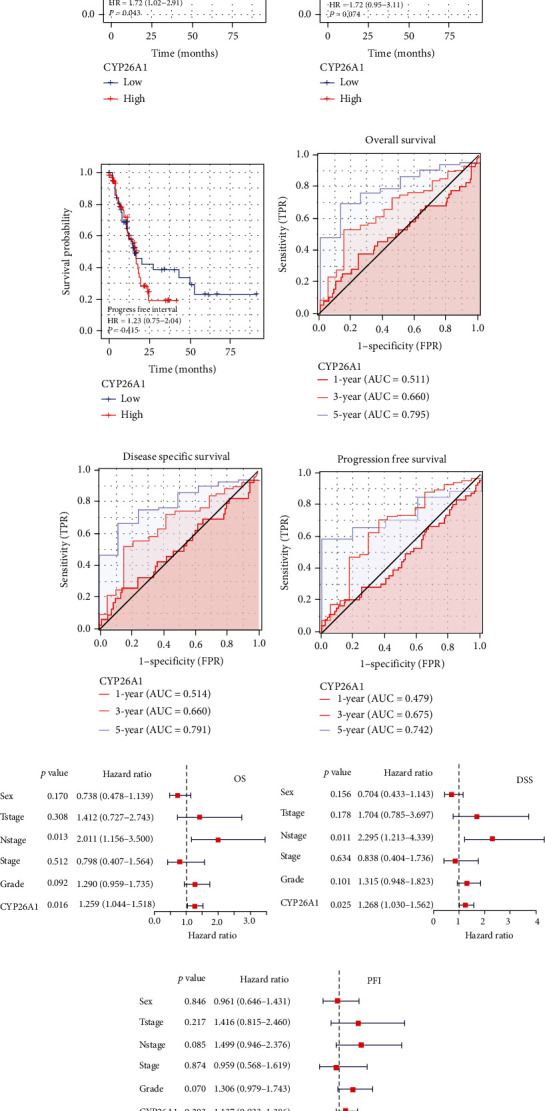
CYP26A1 was a prognosis biomarker of PC. Notes: (a)–(c) Kaplan-Meier survival curves of OS, DSS, and PFI indicated the prognosis value of CYP26A1, respectively. (d)–(f) ROC curves of OS, DSS, and PFI indicated the prognosis value of CYP26A1, respectively. (g)–(i) Multivariate Cox analysis showed that CYP26A1 was an independent prognosis factor of OS, DSS, and PFI.

We further explored the prognosis role of CYP26A1 in PC. Kaplan-Meier survival curves showed that the patients with high CYP26A1 levels had worse OS than those in the low group (Figure 2(a), HR = 1.72, 95%Cl = 1.02–2.91, *P* < 0.05). Despite that the *P* value was not significant, we also found a clear separation in DSS and PFI Kaplan-Meier survival curves (Figures 2(b) and 2(c)). Considering the limitation of sample size, we also considered that CYP26A1 could lead to a worse DSS and PFI prognosis. Furthermore, we investigated the efficacy of CYP26A1 on prognosis prediction. Receiver operating characteristic (ROC) curve showed that CYP26A1 had a satisfactory efficiency in predicting OS, DSS, and PFI of PC patients (Figures 2(d)–2(f), OS: 1-year AUC = 0.511, 3-year AUC = 0.660, 5-year AUC = 0.795; DSS: 1-year AUC = 0.514, 3-year AUC = 0.660, 5-year AUC = 0.791; PFI: 1-year AUC = 0.479, 3-year AUC = 0.675, 5-year AUC = 0.742). Meanwhile, we performed a multivariate cox analysis to explore whether the prognosis influence of CYP26A1 was independent of other clinical features. The result showed that CYP26A1 was an independent prognosis biomarker of OS (*P* = 0.016), DSS, and PFI (Figures 2(g)–2(i)).

### 3.3. CYP26A1 Promotes the Proliferation, Invasion, and Migration of PC Cells

**Figure 3:**
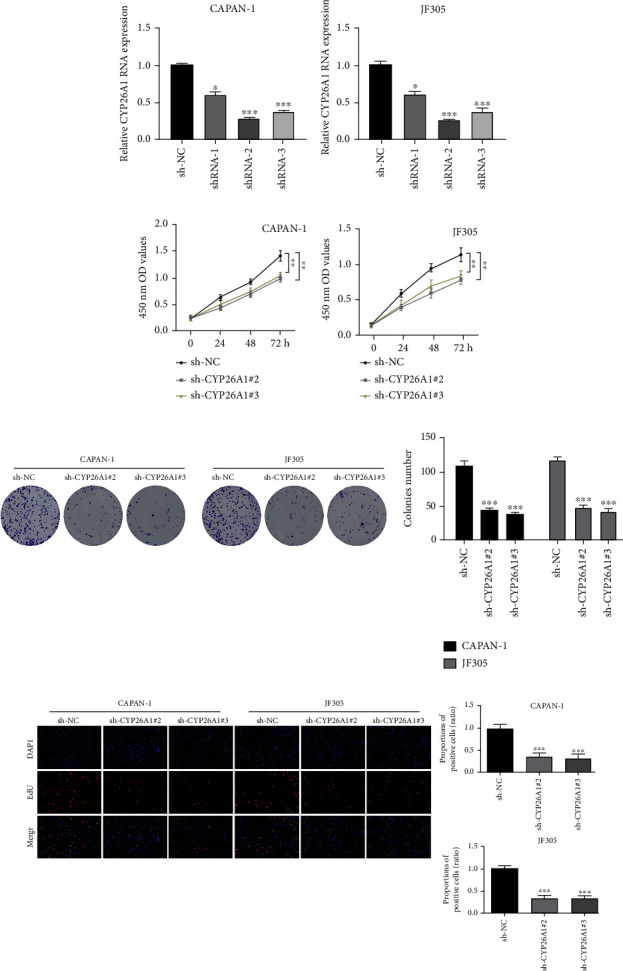
CYP26A1 promotes PC cell proliferation. Notes: (a, b) qPCR assay showed satisfactory knockdown efficiency of CYP26A1 in PC cells. (c, d) CCK8 assay indicated that CYP26A1 could significantly promote PC cell proliferation. (e, f) Colony formation and EdU assay showed that CYP26A1 could remarkably facilitate PC cell proliferation.

**Figure 4:**
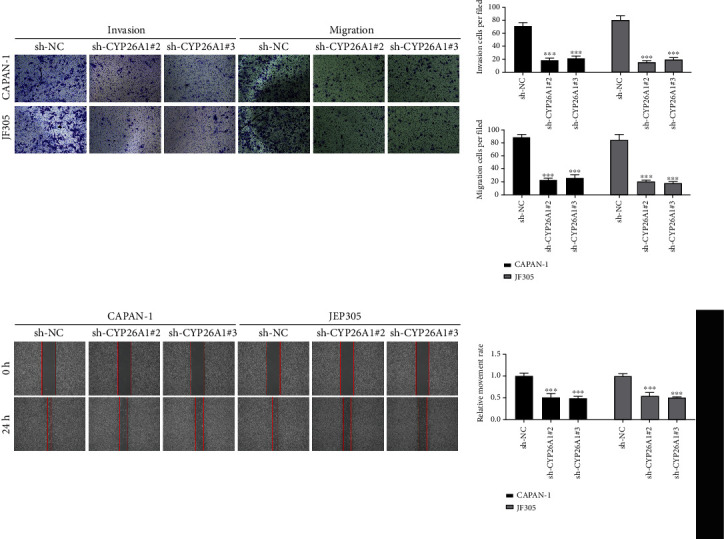
CYP26A1 facilitates PC cell invasion and migration. Notes: (a, b) transwell and wound-healing assay showed that CYP26A1 significantly promotes cell invasion and migration of PC cells.

Considering that CYP26A1 was upregulated in T3-4 patients and metastatic PC cell lines, we further explored the biological role of CYP26A1 through *in vitro* experiments. CAPAN-1 and JF305 cell lines were selected for further experiments for their higher CYP26A1 expression. The knockdown efficiency was validated by qRT-PCR and western blot (Figures 3(a) and 3(b) and Figure S1). The shRNA-2 and shRNA-3 were used for further experiments for their high knockdown efficiency. CCK8 and colony formation assay showed that the knockdown of CYP26A1 could significantly inhibit the proliferation ability of PC cells (Figures 3(c)–3(e)). In addition, the EdU assay indicated that the inhibition of CYP26A1 might remarkably reduce the number of EdU-positive PC cells and led to a lower proliferation rate (Figure 3(f)). Transwell assay showed that CYP26A1 knockdown could hamper the invasion and migration ability of PC cells (Figure 4(a)). A similar trend was also observed in the wound healing assay (Figure 4(b)).

### 3.4. Pathway Enrichment and Immune Infiltration Analysis

**Figure 5:**
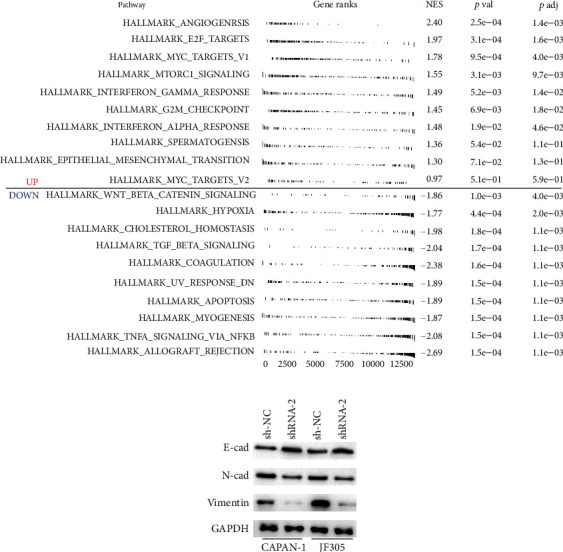
Pathway enrichment of CYP26A1. Notes: (a) GSEA analysis of CYP26A1; (b) Western blot indicated that CYP26A1 could partly activate the EMT pathway.

**Figure 6:**
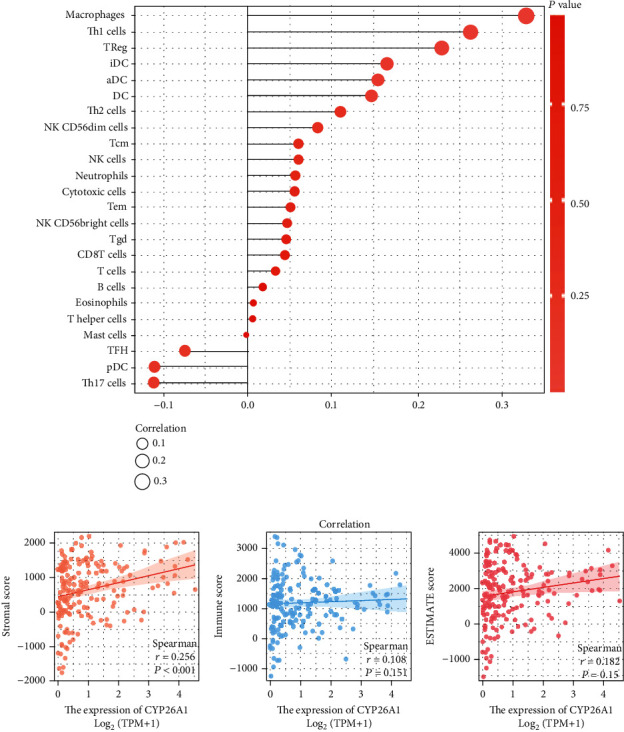
Immune infiltration analysis of CYP26A1. Notes: (a) ssGSEA algorithm was used to quantify the immune infiltration difference in low and high CYP26A1 patients. (b)–(d) Estimate algorithm was used to quantify the immunescore, stromalscore, and estimatescore of PC patients.

We further performed GSEA and immune infiltration analysis to explore the underlying effect of CYP26A1 on PC patients. GSEA analysis showed that the pathways of angiogenesis, E2F target, MYC target, mTORC signaling, G2M checkpoint, and epithelial-mesenchymal transition (EMT) were activated in high CYP26A1 patients (Figure 5(a)). Then, the EMT pathway was selected for validation. Western blot showed that the knockdown of CYP26A1 significantly decreased the protein level of N-cadherin and vimentin, but increased the protein level of E-cadherin, indicating that CYP26A1 could partly activate the EMT pathway (Figure 5(b)). The immune microenvironment has been reported to play an important role in tumor initiation and progression. Therefore, we performed an immune infiltration analysis to investigate the effect of CYP26A1 on the immune microenvironment. The result showed that CYP26A1 was positively correlated with macrophages, Th1 cells, and Treg cells, but negatively correlated with Th17 cells (Figure 6(a)). An estimate package was used to quantify the nontumor cells in the tissue microenvironment. The result showed that risk score had a significantly positive correlation with the stromal cell score, not the immune cell score (Figures 6(b)–6(d)).

### 3.5. CYP26A1 May Affect the Sensitivity of Immunotherapy and Chemotherapy

**Figure 7:**
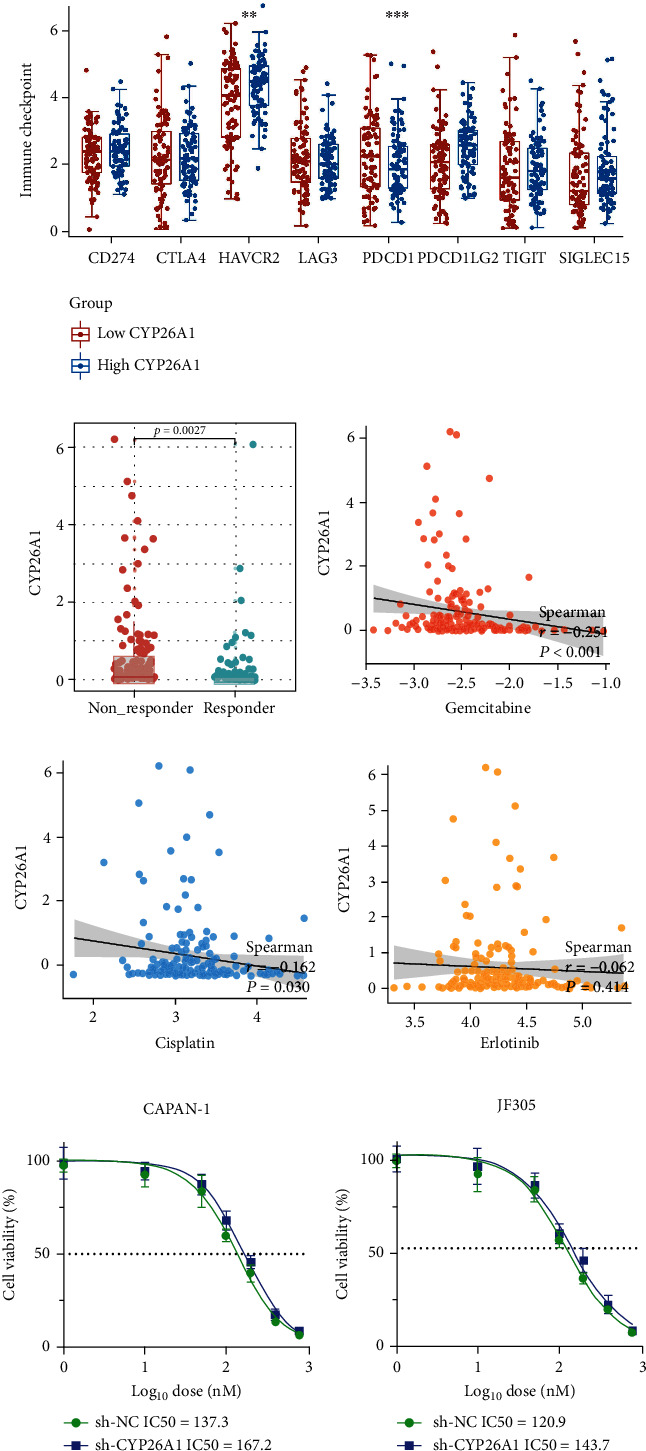
CYP26A1 might be associated with the sensitivity of chemotherapy and immunotherapy. Notes: (a) the correlation between CYP26A1 and multiple immune checkpoints. (b) TIDE analysis showed that the immunotherapy responders had a lower CYP26A1 level than non_responders. (c)–(e) GDSC analysis was performed to explore the correlation between IC50 of chemotherapeutic drugs and CYP26A1 expression. (f, g) Knockdown of CYP26A1 could decrease the sensitivity of PC cells to gemcitabine.

We further explored the association between CYP26A1 and key immune checkpoint molecules. The result indicated that the patients in the high CYP26A1 group tend to have higher HAVCR2 and PDCD1LG2 expression (Figure 7(a)). TIDE analysis showed that the predicted immunotherapy non_responder patients had a higher CYP26A1 level compared with predicted responder patients (Figure 7(b)). Gemcitabine, cisplatin, and erlotinib were the common chemotherapy agents for pancreatic cancer. Thus, we performed drug sensitivity analysis through the GDSC database to identify the underlying effect of CYP26A1 on chemotherapy sensitivity. It seemed that CYP26A1 could remarkably increase the chemotherapy sensitivity of gemcitabine and cisplatin (Figures 7(c)–7(e); IC50, gemcitabine: *r* = −0.251, *P* < 0.001; cisplatin: *r* = −0.162, *P* = 0.03; erlotinib: *r* = −0.0162, *P* = 0.414). Next, gemcitabine was selected for further experimental validation for its most significant *P* value. The result showed that in CAPAN-1 and JF305 cells, the knockdown of CYP26A1 could decrease the sensitivity of PC cells to gemcitabine (Figures 7(f)–7(g), CAPAN-1, sh-NC, IC50 = 137.3, sh-CYP26A1, IC50 = 167.2; JF305, sh-NC, IC50 = 120.9, sh-CYP26A1, and IC50 = 143.7).

## 4. Discussion

PC has an extremely poor prognosis and could significantly impair patient's life [15]. Identification of effective diagnosis and prognosis biomarkers might be beneficial to the treatment of patients and provide new ideas for clinical options.

GSEA analysis showed that the pathways of angiogenesis, E2F target, MYC target, mTORC signaling, G2M checkpoint, and EMT were upregulated in high CYP26A1 patients. Angiogenesis plays an important role in cancer progression. In PC, angiogenesis could significantly promote the growth and metastasis of cancer cells [16]. Meanwhile, Liu et al. showed that the interaction of mTOR and GPX4 could regulate the autophagy-dependent ferroptotic cancer cell death in PC, responsible for its promoting-cancer effect [17]. Meanwhile, Driscoll et al. indicated that the signaling derived by mTORC2 might facilitate PC progression and lead to a worse prognosis [18]. G2M checkpoint is a rate-limiting step in the cell cycle and is closely associated with cell survival [19]. Also, G2M checkpoint was reported to influence the malignant behavior of PC cells in multiple cancers [20, 21]. For example, Duong et al. revealed that BML-275, an AMPK inhibitor, could induce DNA damage, G2/M arrest, and apoptosis in PC cells, thus, exerting its anticancer effect [22]. Li et al. showed that SKA1 could enhance the aggressiveness of PC cells by regulating G2M checkpoints [23]. EMT could confer to cancer cells enhanced plasticity and motility [24]. Recouvreux et al. showed that glutamine depletion could regulate slug to activate the EMT process, resulting in high metastasis potential of PC [25]. In addition, Krebs et al. showed that Zeb1, an EMT-activator, is a key factor for cell plasticity and promotes metastasis in PC [26]. These results indicated that the promoting-cancer effect of CYP26A1 in PC might be achieved through the above antioncogenic pathway.

Immune infiltration analysis showed that CYP26A1 was positively correlated with macrophages, Th1 cells, and Treg cells, but negatively correlated with Th17 cells. Wang et al. showed that hypoxic tumor-derived exosomal miR-301a could mediate M2 macrophage polarization through PTEN/PI3K signaling to facilitate PC metastasis [27]. Nielsen et al. indicated that granulin secreted by macrophages might promote PC metastasis by inducing liver fibrosis [28]. Moreover, the imbalance of Treg/Th17 cells could break the balance between destructive inflammation and defense against tumor cells, further affecting PC cell malignant behavior [29]. Yang et al. showed that the SFRP4 was positively associated with the Foxp3+ Treg cell infiltration, leading to the poor prognosis of PC [30]. Also, Jang et al. demonstrated that tumor-infiltrating Treg cells could induce immunologic tolerance by inhibiting the immunogenicity of tumor-associated dendritic cells, and this crosstalk depends on the interferon-*γ*- (IFN-*γ*-) producing cytotoxic CD8+ T cells [31]. Therefore, the association between CYP26A1 and the immune cells might be partly responsible for its promoting-cancer effect.

To the best of our knowledge, this is the first study focused on the role of CYP26A1 in PC. In this study, we firstly found that CYP26A1 was overexpressed in PC cells and tissue. Clinical correlation showed that CYP26A1 was associated with worse clinical features. Analysis of open-accessed data indicated that CYP26A1 was an independent prognosis biomarker of PC, which also had satisfactory predictive efficiency of OS, DSS, and PFI. *In vitro* experiments showed that CYP26A1 could significantly facilitate the proliferation, invasion, and migration of PC cells. Further, we explored the effect of CYP261A on the biological pathway and immune microenvironment through GSEA and ssGSEA analysis. Gemcitabine, cisplatin, and erlotinib were the common chemotherapy regimen of PC. Therefore, we explored the underlying effect of CYP26A1 on these drugs. The result showed that CYP26A1 could remarkably decrease the chemotherapy sensitivity of gemcitabine and cisplatin. These results indicated that CYP26A1 is a possible therapeutic target of PC with the potential for clinical application.

## Data Availability

Aggregate data are available from the corresponding author on reasonable request.

## Supplementary Materials

Supplementary 1 Figure S1: western blot indicated the knockdown efficiency of CYP26A1 in the protein level.
