# Supplementary figures and images for: CYP26A1 Is a Novel Cancer Biomarker of Pancreatic Carcinoma: Evidence from Integration Analysis and In Vitro Experiments
Source: Dis Markers. 2022 Jun 6;2022:5286820. doi: 10.1155/2022/5286820 (PMC9192288; doi:10.1155/2022/5286820)

Figure S1. Western blot indicated the knockdown efficiency of CYP26A1 in the protein level.

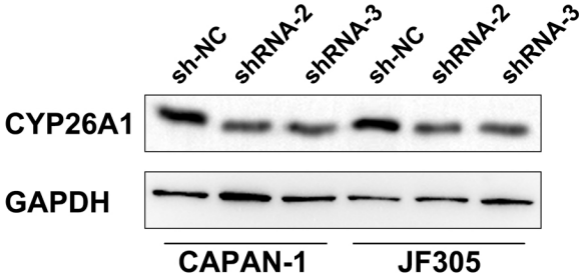

Supplement: Supplementary 1 — Figure S1: western blot indicated the knockdown efficiency of CYP26A1 in the protein level. [file 5286820.f1.pdf]
